# HLA class I alleles frequencies in the Syrian population

**DOI:** 10.1186/s13104-018-3427-1

**Published:** 2018-05-21

**Authors:** Adnan M. Ikhtiar, Batoul Jazairi, Issam Khansa, Ahmad Othman

**Affiliations:** 1 FCM Lab., Molecular Biology & Biotechnology Department, Atomic Energy Commission of Syria (AECS), P.O. Box 6091, Damascus, Syrian Arab Republic; 2 Department of Anatomy, Histology and Embryology, Medicine College, Damascus University, Damascus, Syria

**Keywords:** HLA, Class I, Alleles, Frequency, Haplotypes, Syrian

## Abstract

**Objective:**

The HLA system is known to be the most polymorphic genetic loci in humans. Distribution and frequencies of HLA alleles are highly variable among different human ethnic groups. The HLA system has an important role in disease susceptibility and resistance, especially in autoimmune diseases and cancer. This study is the first report about HLA genetic variability and haplotypes among Syrians. Frequency of the HLA class I (A, B and C) alleles was determined in 105 healthy unrelated Syrian individuals from different regions in Syria. We also studied the associated haplotypes frequencies. Alleles frequencies were compared with those reported for other populations.

**Results:**

Fifty-eight HLA class I alleles were observed in Syrians including 15 for HLA-A, 28 for HLA-B and 15 for HLA-C. We observed 37 HLA-A/C haplotypes, 32 B/C, and 31 A/B haplotypes. The most frequent haplotypes were A*01/C*04, A*02/C*07, A*02/B*35, and B*35/C*04. In conclusions, our preliminary study suggests a high variability in HLA class I alleles in the Syrian population. This study also gives a general reference database about the genetic pool distribution of HLA class I alleles among Syrians and can be consulted for HLA related diseases.

## Introduction

Major histocompatibility complex (MHC) consists of genetic loci that involved in foreign organs rejection and encode highly polymorphic cell surface molecules. The human MHC is called the human leukocyte antigens (HLA) system because these antigens were first identified and characterized using alloantibodies against leukocytes [1]. The human MHC (HLA), maps to the short arm of chromosome 6 (6p21) and spans approximately 3600 kilo bases of DNA [2]. The HLA system consists of two classes: class I region contains HLA-A, HLA-B and HLA-C genes and class II region contains HLA-DR, HLA-DQ and HLA-DP [3].

Association between HLA and certain diseases has been well studied; several diseases reported to occur more frequently in individuals with particular HLA genotypes [4–8]. These diseases include a broad spectrum of immune-mediated diseases involving all major organ systems, certain malignancies, some infectious diseases and more recently, adverse reactions to particular drugs [4, 8]. HLA associations are widely studied across the human populations worldwide and are found to be important in the prediction of disease susceptibility, resistance and for evolutionary maintenance of genetic diversity [5, 9]. HLA genes frequency distribution in different human ethnic groups can help in determining the ancestry, human migrations and admixtures between different human populations [10].

The Syrian population is genetically diverse due to the geographical location of Syria in the Mediterranean basin, history of invasions and migrations from different nations. Syria has a mixture of cultural, religious and ethnic groups which may include Egyptians, Hittites, Sumerians, Mitanni, Assyrians, Babylonians, Canaanites, Phoenicians, Arameans, Amorites, Persians and the Greeks [11, 12]. Syria was ruled by Arab Umayyad, Hamdanids, Byzantines, and Fatimids. In the followed centuries, Syria was held by the Crusaders, Mongols, Egyptians, Mamluks, and Turko-Mongol from central Asia [13]. Syria was occupied by the Ottoman Empire from 1516 till 1918 [14]. After World War I, Syria became under the direct dominance of France until independence in 1946 [15]. All those invaders gave Syria its ethnic diversity.

The aim of our research was to study the diversity of HLA class I-A, B and C alleles among Syrians and to establish a preliminary reference data set for other studies such as association between HLA and chronic diseases in Syria.

## Main text

### Methods

One hundred and five unrelated healthy Syrian adults from different areas were randomly chosen while donating blood in the Damascus Blood Bank.

DNA was prepared from anticoagulated whole blood using the modified salting out method [16, 17]. HLA typing of HLA-A, B, C alleles were performed using PCR sequence-specific priming (SSP) technique (One Lambda, Inc., USA) per manufacturer^’^s instruction.

The allele frequencies (AF) were calculated by direct counting method (Total number of copies of the allele in the population sample/2n) [18]. Gene frequencies (GF) were determined by the following equation: $\left( {{\text{GF}} = 1- \sqrt { 1- {\text{AF}}} } \right)$ [19]. The haplotypes frequencies were calculated based on Hardy–Weinberg law [20].

### Results

A total of 58 HLA alleles were found in the study population: 15 HLA-A, 28 HLA-B and 15 HLA-C alleles. HLA-A, B and C alleles frequencies are given in Table 1. The most common alleles for HLA class I A locus were: A*02, A*24, A*01, A*03; for B locus were: B*35, B*51, B*44, B*52 and for C locus were: C*04, C*07, C*12, C*06.

**Table 1:** HLA-A, -B, -C alleles and genes frequencies in Syrian population (n = 105)

Alleles n = 105	AF (%)	GF (%)	Alleles n = 105	AF (%)	GF (%)	Alleles n = 105	AF (%)	GF (%)
A*01	12.9	6.7	B*07	4.3	2.2	C*01	3.8	1.9
A*02	19	10	B*08	4.8	2.4	C*02	2.4	1.2
A*03	12.9	6.7	B*13	3.3	1.7	C*04	22.9	12.2
A*11	3	1.5	B*14	6.2	3.1	C*05	1.4	0.7
A*23	3	1.5	B*15	2.4	1.2	C*06	10.5	5.4
A*24	13.1	6.8	B*18	4.8	2.4	C*07	21.4	11.3
A*26	3.6	1.8	B*27	2.4	1.2	C*08	6.2	3.1
A*29	3	1.5	B*35	18.6	9.8	C*09	1.4	0.7
A*30	9	4.6	B*37	1	0.5	C*10	1	0.5
A*31	5.2	2.6	B*38	5.7	2.9	C*12	15	7.8
A*32	1	0.5	B*39	1	0.5	C*14	1	0.5
A*33	5.2	2.6	B*40	2.4	1.2	C*15	6.2	3.1
A*36	1	0.5	B*41	1	0.5	C*16	3.4	1.7
A*68	7.1	3.6	B*42	0.5	0.3	C*17	1.5	0.8
A*69	0.5	0.3	B*44	7.6	3.9	C*18	1.9	1
			B*45	1.9	1			
			B*47	1	0.5			
			B*49	5.7	2.9			
			B*50	1.9	1			
			B*51	8.1	4.1			
			B*52	7.1	3.6			
			B*53	3	1.5			
			B*55	2.4	1.2			
			B*57	0.5	0.3			
			B*58	1.4	0.7			
			B*70	0.5	0.3			
			B*73	0.5	0.3			
			B*8201	0.5	0.3			

Table 2, describe the HLA class I haplotypes (A/B, B/C, A/C and A/B/C) found in Syrian population. Tables 3 and 4, respectively compare allele frequencies of the A and B loci in the studied Syrian population with those frequencies reported for other human populations from Jordan, Lebanon, Turkey, Russia, Russia Tatar, Iran, China, Emirates, Morocco, France, Greece, Macedonia, Mongolia and Sudan.

**Table 2:** HLA class I haplotypes (A/B, B/C, A/C and A/B/C) found in Syrian population (n = 105)

Haplotypes A/C	HF %	Haplotypes A/B	HF %	Haplotypes B/C	HF %	Haplotypes A/B/C	HF %
A*01/C*07	0.06	A*01/B*08	0.013	B*08/C*04	0.025	A*02/B*49/C*07	0.024
A*01/C*18	0.04	A*01/B*35	0.05	B*08/C*06	0.04	A*24/B*49/C*07	0.024
A*01/C*04	0.19	A*01/B*38	0.02	B*08/C*07	0.08	A*11/B*52/C*12	0.0002
A*01C*05	0.004	A*01/B*49	0.02	B*08/C*18	0.008	A*03/B*52/C*12	0.0032
A*02/C*07	0.2	A*01/B*55	0.007	B*13/C*06	0.008		
A*02/C*08	0.08	A*02/B*07	0.02	B*13/C*07	0.02		
A*02/C*04	0.09	A*02/B*13	0.013	B*18/C*01	0.004		
A*02/C*17	0.007	A*02/B*18	0.02	B*18/C*12	0.02		
A*02/C*16	0.014	A*02/B*35	0.07	B*35/C*07	0.08		
A*02/C*18	0.01	A*02/B*42	0.003	B*35/C*09	0.006		
A*02/C*12	0.06	A*02/B*44	0.03	B*35/C*04	0.09		
A*03/C*04	0.06	A*02/B*51	0.03	B*35/C*18	0.01		
A*03/C*07	0.06	A*03/B*13	0.01	B*38/C*12	0.02		
A*03/C*01	0.01	A*03/B*35	0.05	B*44/C*04	0.04		
A*03/C*12	0.04	A*03/B*55	0.007	B*44/C*06	0.02		
A*24/C*07	0.06	A*03/B*61	0.004	B*44/C*18	0.002		
A*24/C*04	0.06	A*03/B*65	0.014	B*45/C*04	0.01		
A*24/C*12	0.04	A*24/B*35	0.05	B*49/C*07	0.03		
A*26/C*12	0.01	A*24/B*44	0.02	B*50/C*04	0.01		
A*29/C*04	0.01	A*24/B*45	0.006	B*50/C*12	0.007		
A*30/C*04	0.04	A*30/B*18	0.01	B*51/C*04	0.04		
A*30/C*07	0.02	A*30/B*49	0.011	B*51/C*01	0.007		
A*30/C*12	0.01	A*31/B*08	0.005	B*51/C*07	0.02		
A*31/C*07	0.02	A*31/B*44	0.009	B*52/C*12	0.02		
A*31/C*01	0.005	A*31/B*51	0.01	B*52/C*08	0.009		
A*31/C*06	0.01	A*31/B*52	0.008	B*55/C*07	0.01		
A*31/C*08	0.006	A*31/B*64	0.001	B*55/C*16	0.002		
A*32/C*04	0.006	A*68/B*35	0.03	B*57/C*07	0.003		
A*32/C*12	0.004	A*68/B*51	0.012	B*58/C*07	0.007		
A*33/C*04	0.03	A*68/B*53	0.005	B*63/C*07	0.01		
A*33/C*07	0.02	A*68/B*65	0.008	B*65/C*08	0.006		
A*33/C*08	0.007	A*02/B*49	0.02				
A*68/C*04	0.04	A*24/B*49	0.02				
A*68/C*08	0.01	A*03/B*52	0.02				
A*68/C*12	0.02	A*11/B*52	0.005				
A*68/C*18	0.003						
A*11/C*12	0.01

**Table 3:** Locus A alleles frequencies in the Syrian population compared to other populations

Allele	Syria n = 105	Jordan n = 15141	Lebanon n =1994	Turkey n =228	Russia n =2650	Russia Tatar n =135	Iran n =15600	China n =26266	Emirates n = 373	Morocco n = 96	France n =1000	Macedonia n =172	Greece n =246	Mongolia n = 85	Sudan n =46
A*01	0.129	0.15	0.23	0.066	0.114	0.118	0.116	0.024	0.062	0.148	0.1266	0.137	0.0917	0.08	0.0444
A*02	0.19	0.213	0.405	0.219	0.288	0.274	0.171	0.313	0.252	0.262	0.2633	0.256	0.2536	0.265	0.2775
A*03	0.129	0.088	0.25	0.109	0.134	0.159	0.108	0.019	0.091	0.067	0.1465	0.119	0.0873	0.06	0.0444
A*11	0.03	0.041	0.105	0.061	0.063	0.074	0.09	0.226	0.102	0.067	0.0679	0.076	0.0566	0.1	0
A*23	0.03	0.039	0.065	0.053	0.028	0.019	0.02	0.002	0.031	0.041	0.0285	0.035	0.0207	0.015	0.0217
A*24	0.131	0.104	0.205	0.213	0.096	0.107	0.134	0.173	0.052	0.073	0.1012	0.163	0.1467	0.195	0.011
A*26	0.036	0.044	0.07	0.135	0.049	0.063	0.057	0.026	0.074	0.014	0.0318	0.067	0.0371	0.06	0.0217
A*29	0.03	0.03	0.065	0.015	0.017	0.007	0.025	0.007	0.016	0.034	0.0555	0.012	0.0289	0.01	0.011
A*30	0.09	0.087	0.08	0.013	0.028	0.03	0.052	0.06	0.05	0.101	0.0237	0.017	0.0351	0.02	0.1925
A*31	0.052	0.017	0.015	0.029	0.02	0.022	0.015	0.036	0.028	0.006	0.0339	0.02	0.0207	0.085	0.011
A*32	0.01	0.035	0.055	0.028	0.031	0.022	0.056	0.009	0.038	0.027	0.0345	0.032	0.0694	0.02	0.011
A*33	0.052	0.035	0.04	0.011	0.023	0.022	0.038	0.089	0.061	0.027	0.014	0.012	0.033	0.06	0.011
A*36	0.01	0.001	0.04	0	0	0	0	0	0.004	0	0	0	0	0	0.011
A*68	0.071	0.048	0	0.035	0.038	0.044	0.04	0.005	0.084	0.093	0.0415	0.038	0.0351	0.015	0.1151
A*69	0.005	0.048	0	0	0.003	0	0.004	0.007	0.003	0	0.0016	0	0.006	0	0.056

**Table 4:** Locus B alleles frequencies in the Syrian population compared to other populations

Allele	Syria n= 105	Jordan n= 15141	Lebanon n =1309	Turkey N =228	Russia n =2650	Russia Tatar n =135	Iran n =15600	China n =26266	Emirates n = 373	Morocco n = 96	France n =1000	Macedonia n =172	Greece n =246	Mongolia n = 85	Sudan n =46
B*07	0.043	0.032	0.085	0.039	0.12	0.107	0.04	0.018	0.024	0.09	0.1328	0.064	0.0248	0.055	0.011
B*08	0.048	0.023	0.045	0.035	0.067	0.037	0.03	0.007	0.086	0.058	0.0965	0.067	0.0413	0.035	0.011
B*13	0.033	0.028	0.065	0.044	0.066	0.107	0.037	0.105	0.015	0.011	0.015	0.015	0.0289	0.04	0.056
B*14	0.062	0.041	0.043	0	0.027	0.004	0.028	0.0009	0.015	0.047	0.0467	0.009	0.0371	0	0
B*15	0.024	0.049	0.029	0.035	0.054	0.019	0.032	0.15	0.054	0.068	0.0726	0.018	0.0351	0.115	0.3239
B*18	0.048	0.046	0.075	0.031	0.084	0.074	0.049	0.003	0.048	0.042	0.0456	0.166	0.1444	0.005	0.033
B*27	0.024	0.017	0.03	0.028	0.051	0.048	0.017	0.031	0.006	0.005	0.0389	0.041	0.0207	0.025	0.033
B*35	0.186	0.141	0.355	0.14	0.098	0.122	0.156	0.043	0.111	0.053	0.0783	0.148	0.1421	0.075	0.033
B*37	0.01	0.009	0.01	0.004	0.01	0.011	0.008	0.01	0.02	0	0.0114	0.02	0.008	0.035	0.0217
B*38	0.057	0.042	0.07	0.033	0.044	0.044	0.047	0.027	0.011	0.026	0.013	0.052	0.0248	0.02	0
B*39	0.01	0.014	0.005	0.011	0.02	0.041	0.007	0.028	0.012	0.021	0.0228	0.017	0.0289	0.01	0
B*40	0.024	0.022	0.035	0.058	0.046	0.056	0.036	0.159	0.064	0.058	0.0591	0.035	0.0268	0.145	0.033
B*41	0.01	0.057	0.105	0.015	0.032	0.03	0.036	0.0008	0.014	0.026	0.0109	0.012	0.0207	0	0.056
B*42	0.005	0.008	0	0	0	0	0.001	0.0001	0.008	0.005	0.001	0	0	0	0.0791
B*44	0.076	0.061	0.135	0.134	0.101	0.067	0.041	0.025	0.023	0.053	0.1618	0.078	0.0672	0.04	0
B*45	0.019	0.021	0.035	0.004	0.002	0	0.003	0.002	0.01	0.074	0.0067	0.003	0.002	0	0
B*47	0.01	0.006	0.01	0	0.004	0	0.002	0.0001	0	0	0.0026	0	0.012	0.005	0.0911
B*49	0.057	0.067	0.085	0.004	0.017	0.033	0.026	0.0008	0.008	0.058	0.0228	0.015	0.0207	0.01	0.011
B*50	0.019	0.066	0.04	0.05	0.014	0.029	0.055	0.004	0.094	0.053	0.0109	0.006	0.01	0.02	0
B*51	0.081	0.099	0.155	0.158	0.04	0.059	0.121	0.068	0.156	0.074	0.0648	0.148	0.1544	0.125	0.0217
B*52	0.071	0.046	0.07	0.017	0.031	0.026	0.054	0.027	0.052	0.037	0.0067	0.023	0.012	0.025	0
B*53	0.03	0.025	0	0	0.002	0	0.008	0.0004	0.022	0.042	0.0052	0.003	0.0166	0	0.0444
B*55	0.024	0.019	0.045	0.071	0.01	0.007	0.03	0.03	0.011	0.005	0.0218	0.026	0.0309	0.025	0
B*57	0.005	0.029	0.015	0.013	0.03	0.019	0.014	0.01	0.011	0.021	0.0306	0.002	0.0227	0.02	0.011
B*58	0.014	0.025	0.025	0.008	0.01	0.019	0.017	0.068	0.05	0.063	0.0104	0.009	0	0.07	0.0672
B*70	0.005	0	0	0	0	0	0		0	0	0	0	0	0.005	0
B*8201	0.005	0	0	0	0	0	0	0	0	0	0	0	0	0	0

### Discussion

Our preliminary results suggest a high diversity in HLA class I alleles among Syrians. Our findings about HLA-A locus alleles frequencies were consistent with those found by other research group on HLA-A alleles in the Syrian population genotyped by sequence-based typing [21]. The allele with the maximal frequency was A*02 as in most human populations throughout the world [22]. Among the high frequent alleles in our cohort (Table 1), the A*02, A*24 and A*03 were also the most frequent alleles in Iranians, Greeks, Russians, Russians Tatars, French, and Macedonians. HLA-A*24 was the second most frequent HLA-A allele in the studied Syrian population, this allele was also the second most frequent HLA-A allele in Southeastern European populations, including Italians [23] and Albanians [24], whereas the third most frequent HLA-A allele in studied Syrian population. The (HLA-A*01, HLA-A*03) are the second most frequent in Western and Central Europe populations [25–30]. Whereas, the HLA-A*26 allele which is highly frequent in Turks, has a moderate frequency in the studied Syrian population. The HLA-A*11 allele which has a high frequency in Emiratis, Chinese, and Mongolians, was in moderate frequency in the studied Syrian population. The HLA-A*33 allele which is in a high frequency in Chinese, was relatively in high frequency in the studied Syrian population. The HLA-A*30 and HLA-A*68 alleles which are in high frequency in Sudan, were also, relatively, in a high frequency in the studied Syrian population. In contrast, the HLA-A*43 allele which is frequent in the African populations was absent in the studied Syrian population. The HLA-A*36 allele which is common in Africans was in low frequency in the studied Syrian population [31, 32]. The HLA alleles: A*25, A*34, A*66, A*74, and A*80 were absent in Syrian population.

For the B locus, the four most frequent alleles (above 5%) in our cohort HLA-B*35, -B*51, -B*44 and -B*52 (Table 1) were previously reported in neighboring countries: Greece, France, Macedonia, and Russia [33–36]. The B*35 allele which is the most frequent allele in our cohort, is also in high frequency in Iranians, and Russians Tatars [37]. The HLA-B*51 allele, which is the second high frequent allele in our cohort, is in highly frequent in Turks, Iranians, Emiratis, Moroccans, Greek, Macedonians, and Mongolians [10, 35, 36, 38–41]. The HLA-B*52 allele which comes in the fourth order in frequency in the studied Syrian population, is also in high frequency in the Iranians [39]. The HLA-B*07 allele which is the most frequent in Moroccans and Russians [33, 40], was in moderate frequency in the studied Syrian population. The HLA-B*18 allele which is in high frequency in Greek, Russians, Russians Tatars, and Macedonians [33, 35–37], was in moderate frequency in the studied Syrian population. The HLA-B*13 allele, which is in high frequency in Russians Tatars and Chinese, was moderately frequent in the studied Syrian population (Table 3). The alleles in moderate frequencies in our cohort (B*08, B*40, B*15), were highly frequent in the French (HLA-B*08) [34], Emiratis [10], Chinese and Mongolians (HLA-B*40) [41, 42] and in Sudanese and Mongolians (HLA-B*15) [36, 43]. The B*47, B*42, and B*48 alleles, which are absent in the studied Syrian population, are in high frequency among the Sudanese [43]. Other HLA alleles: B*46, B*48, B*54, B*56, B*59, B*67, B*73, and B*81 were absent in the studied Syrian population, (Table 3).

This study also demonstrated that the most frequent alleles for HLA-C locus were HLA-C*04, HLA-C*07, HLA-C*12 and HLA-C*06 (Table 1).

The distribution of allele groups in the studied Syrian population showed a high similarity with HLA allele groups in European human populations, such as: A*02, A*24, A*1, A*3, B*35, B*51, B*44 and B*52 [32]. This similarity prevalence of HLA alleles frequency in the Syrian population with those in human populations from the Eastern Mediterranean (Lebanon, Jordan) [44] and in human populations in north Mediterranean (Greece, Macedonia, Italy, Russia Tatar and France), may be explained by the fact that throughout history the East Region of the Mediterranean (Syria, Lebanon, Palestine and Jordan), was endured many waves of invasions from the north and the east, which originated from the Greeks, Romans, Tatars, Ottomans and Persians [45], resulting in genetic admixtures between local residents and invaders across human history in the Middle East region.

Among the most frequent two-locus haplotypes in Syrian population, the B*35/C*04 haplotype was the most frequent, while the B*08/C*07 haplotype, which is the most frequent in other human populations, was the second most frequent in our population. The identified frequent haplotypes in our studied group were also predominant in some Mediterranean populations, such as: Lebanese [46], Iranians [39] and Europeans [47].

### Conclusion

This preliminary study is the first report about the frequencies and distribution of HLA-A, -B and -C alleles among Syrians. By comparing HLA-A alleles frequencies, (The: A*02, A*03, A*24 and A*01 in particular), in the studied Syrian population with those reported for other human populations we find that Syrians are genetically closer to Jordanians, Lebanese, Turks, Russians, Tatars, Iranians and the French. HLA-B alleles frequencies (particularly the B*35, B*44 and B*51 alleles) gets Syrians closer to Jordanians, Lebanese, Turks, Russians, and French.

In general the Syrian population genetically closer to neighboring human populations, (Jordanians, Lebanese, and Turks), and to Europeans in the north of the Mediterranean. Our results also shows that the Syrians are genetically far from human populations form the Arabian Peninsula [48] and North Africa [49, 50], and very far from the Chinese and other African human populations.

## Limitations

This study also gives a general reference database about the genetic pool distribution of HLA class I alleles among Syrians and can be consulted for HLA related diseases. The results can also be consulted in anthropological studies. It also advocates for further studies to assess the association of HLA-A, -B and -C alleles with major chronic diseases in the Syrian population.

## Abbreviations

HLA: human leukocyte antigen
PCR-SSP: single specific primer-polymerase chain reaction
MHC: major histocompatibility complex
